# A Taxonomy of Ethical, Legal and Social Implications of Wearable Robots: An Expert Perspective

**DOI:** 10.1007/s11948-020-00268-4

**Published:** 2020-09-29

**Authors:** Alexandra Kapeller, Heike Felzmann, Eduard Fosch-Villaronga, Ann-Marie Hughes

**Affiliations:** 1 Department of Thematic Studies: Technology and Social Change, Linköping University, Linköping, Sweden; 2 Department of Philosophy, National University of Ireland, Galway, Ireland; 3 Center for Law and Digital Technologies, Leiden University, Leiden, The Netherlands; 4 Faculty of Health Sciences, University of Southampton, Southampton, UK

**Keywords:** Exoskeleton, Wearable robots, Ethical, legal, and societal (ELS) aspects, ELSI, Taxonomy

## Abstract

Wearable robots and exoskeletons are relatively new technologies designed for assisting and augmenting human motor functions. Due to their different possible design applications and their intimate connection to the human body, they come with specific ethical, legal, and social issues (ELS), which have not been much explored in the recent ELS literature. This paper draws on expert consultations and a literature review to provide a taxonomy of the most important ethical, legal, and social issues of wearable robots. These issues are categorized in (1) wearable robots and the self, (2) wearable robots and the other, and (3) wearable robots in society.

## Introduction

Wearable robots (WRs), including robotic exoskeletons and orthoses, are an emerging technology designed to augment, train, or supplement motor functions (Greenbaum 2015a). These devices are integrated parts of human motor functioning, and are constructed of typical hardware (actuators and sensors) and software (control algorithms) components (CA16116 2017). Usually worn over clothing, they are ‘mechanical devices that are essentially anthropomorphic in nature, ‘worn’ closely fitting the user’s body, and work in concert with the operator’s movements’ (Dollar and Herr 2008; Herr 2009). However, their interaction with humans is not exclusively physical; it ‘also includes cognitive aspects … [insofar as] control of functions is typically shared by human and machine’ (CA16116 2017; Pons 2010). Given these intimate connections between WRs and their users, WRs are likely to have considerable impact on the users and their social environment and raise particular questions about data protection, safety, responsibility, ableism, and identity.

This paper sets the scene for a more comprehensive consideration of such ethical, legal and social (ELS) issues. Building on expert consultations and a literature review, our aim in this paper is to provide a taxonomy of the most relevant ELS issues pertaining to WRs. Although some of these ELS concerns are shared with other types of robots and information technologies, WRs’ unique combination of features raises specific issues. For example, wearable computing, such as fitness trackers, smartwatches, or head-mounted displays, are also body-borne devices and ‘inextricably intertwined’ with humans (Mann 2012) but lack the WRs’ direct impact on motor functions. Social robots are external devices that interact with users socially and are not worn, and prostheses replace, rather than support, limb functions. However, it should be noted that prostheses have been understood as WRs as well (Bergamasco and Herr 2016, p. 1876).

The need to investigate the particular ELS issues of WRs also stems from their wide range of potential applications. While rehabilitation robots aim to supplement body functions to reach a basic level, enhancement robots aim to augment body functions beyond what may be considered an ‘average’ level (Herr 2009). Although the boundaries between rehabilitation and enhancement are fluid, rehabilitation is the primary goal in healthcare, whereas enhancement is the primary goal in the industry, military and leisure/sports applications of WRs. The design and implementation of these devices in different domains need guidance and regulation, not just with regard to technical and safety aspects, but also concerning personal, interpersonal, and broader societal effects.

While there is a developing body of literature on ELS issues in WRs, it is only in its early stages (e.g. Bulboacă et al. 2017; Sadowski 2014) and covers relevant issues unevenly. Literature concerned with human-centred and user-centred design in the field, in particular, often has a technical focus and lacks deeper reflection on ELS issues (e.g. Contreras-Vidal et al. 2015; Meyer et al. 2019; Power et al. 2019). Current guidance and regulation in Europe primarily consist of standards for industrial and care robots (International Organization for Standardization 2014), data protection law (General Data Protection Regulation (GDPR) 2016), medical device regulation (MDR), and product safety laws. The blurry intertwinement between public and private regulatory bodies together with gaps in existing regulation stand in the way of the development of a coherent regulatory approach to addressing ELS challenges (Fosch Villaronga and Golia 2019).

A taxonomy of ELS issues related to WRs can serve many purposes. In addition to contributing to the academic debate on ELS issues in healthcare and enhancement technologies, it can guide the integration of ELS considerations in the design process, addressing concerns for developers, users, and societal stakeholders. A comprehensive view of these ELS challenges is also valuable for policymaking, as it highlights areas of concern that require further exploration, where existing ethical or legal frameworks might apply, or where regulatory action might be needed (Fosch-Villaronga and Heldeweg 2018). Such regulatory action is particularly important for WRs: a review of WRs currently available or in development shows that the field is rapidly expanding (Bergamasco and Herr 2016) and the exoskeleton market size has been projected to reach USD 4.2 billion by 2027, growing at a compound annual growth rate (CAGR) of 26.3% until 2027 (Grand View Research 2020).

Our expert consultations and literature review resulted in twelve ELS issues, sorted into three clusters, covering individual, relational, and societal aspects. While we address WRs across different domains, we excluded military applications from our considerations and instructed experts accordingly. This exclusion is due to the restriction of COST funding to “peaceful purposes” only. COST (2019) states “any funding of activities related to sensitive technology development, armament or defence-oriented research should be avoided” (p. 3), so no exploration was conducted into how WRs may fit into existing ethical frameworks for military robots (e.g. Amoroso and Tamburrini 2018; Lucas 2014; Sharkey 2019; Sparrow 2016).

This contribution seeks to highlight the importance of identifying and addressing ELS issues during the development of WRs to ensure that these technologies are designed and employed in a way that achieves benefits to users and society while being alert to potential risks.

## Methodology

Three consultations with experts, i.e. philosophers, social scientists, data protection lawyers, medical professionals, and engineers, were held between 2017 and 2018 under CA16116:

- Portugal, 2017: 30 experts and students from engineering, healthcare and rehabilitation, philosophy, and the regulatory field
- Netherlands, 2018: 30 experts in robot ethics, wider ELS issues regarding robots, and the philosophy of technology
- Italy, 2018: 20 experts in engineering, philosophy of technology, robot ethics, technology law, and others

In all workshops, participants were asked to brainstorm ELS issues on individual post-it notes. The session facilitators pre-clustered them, introduced and then displayed them publicly in the second phase. After participants had a chance to familiarize themselves with this overview, in the third phase, they were asked to discuss in groups and select three issues they deemed most important, with a plenary discussion concluding the session. Due to time constraints, the third workshop skipped phase two, and a brief plenary presentation followed the discussion groups.

All notes from the three sessions were transcribed into a list, and their frequency of occurrence and the group selections were separately noted. Similar issues were clustered together, and a mind-map of issues was produced, enabling the identification of common themes. A non-systematic literature research on these themes complemented and supported the experts’ perspectives. The primary goal of the analysis was not a detailed representation of the opinions identified in each workshop, but the creation of a map of ELS concerns that could provide structure and context to the concerns identified in those discussions.

## Results

### Wearable Robots and the Self

#### Body and Identity Impacts

Like other technologies that are intimately linked to the body, WRs affect a user’s self-perception and identity; they are likely to change not only their functional abilities but also how their self is experienced (Barfield and Williams 2017; Breen 2015). Other work has demonstrated how assistive technologies, especially wheelchairs, can become a part of their users’ identities, necessitating a conceptual re-evaluation of the body (Barfield and Williams 2017; Palmerini et al. 2014). Both the smooth integration of a WR into a person’s body schema and the experience of friction arising from limitations of the WR that do not correspond to the user’s movement intentions may impact on the perception of their self. Experts raised concerns that WR users, similar to prosthesis users (Murray and Fox 2002), might struggle with a new body image, e.g., feeling partly machine-like, and question whether and how far they own their bodies and movements. Further, relying on the WR for fundamental activities such as walking, grasping, or working results in dependence on the technology with potential implications for a person’s self-understanding if the technology is withdrawn (Bissolotti et al. 2018; Greenbaum 2015a). The technology’s incorporation into identities also raises questions concerning data transferability from WRs. Some experts highlighted that if WRs become finely adjusted to individual parameters, disruptions to users’ body experience when changing WRs might be alleviated by ensuring transferability of such data to new devices.

#### The Experience of Vulnerability

Users of WRs may experience different vulnerabilities. Vulnerability as the ‘capacity to suffer that is inherent in human embodiment’ (Mackenzie et al. 2014, p. 4) is relevant in rehabilitation contexts, where the user’s underlying condition may be perceived as a vulnerability (e.g., impaired mobility with associated health and social risks), which the use of WRs could potentially ameliorate. At the same time, the use of the WR may itself cause vulnerabilities for users. Experts highlighted especially the vulnerabilities arising from dependence on WRs for users with mobility impairments, both through dependency on potentially limited functions or operational risks of the WR, but also due to potential consequences of withdrawing the WR. Further, workers can be vulnerable to exploitation in industrial, logistical or care settings, where employers might demand WR use, leading to changing work conditions and potentially increasing performance pressures on workers.

The ethical discussion of vulnerability highlights the risk of essentializing vulnerability, and the importance of the social context of vulnerabilities (Luna 2009, 2019; Luna and Vanderpoel 2013). For example, while a mobility disability constitutes a potential vulnerability, other contextual factors will affect the resultant impact upon a person. If they can access a high level of care and social support, and there is a high level of physical accessibility, the vulnerability arising from mobility may be compensated. In contrast, if those factors are absent, this may result in “cascading vulnerabilities” (Luna 2019).

#### Agency, Control and Responsibility

The use of WRs also raises questions of control and agency. Usually, WR users need to engage in a period of training to adapt to the shared movement control between the user and the WR. Especially in the early stages, users might feel that they are not entirely in control of the assemblage of their body and the machine. Not having confidence in their movements can lead to an experienced decrease of agency: ‘Am I walking in the suit, or is the suit walking me?’ (Cornwall 2015). A prominent concern is the potential for the WR to lead to unintended and potentially risky movements, generating ‘destructive forces whose controlled output behavior may not always be in agreement with the user’s intent’ (Tucker et al. 2015). Users might want to perform movements that the WR is not programmed for, or might not want to perform movements in the manner that the WR automatically generates. While integration of the WR into the user’s body schema occurs, the residual friction that remains affects a WR user’s sense of control.

The notions of agency and control are generally considered to be closely linked to responsibility (Fischer and Ravizza 2000). While in the field of robot ethics and robot law, the issue of responsibility for robots’ actions has been explored extensively (Lokhorst and van den Hoven 2011; Matthias 2004; Nyholm 2018; Sparrow 2007), in the case of WRs the question of responsibility has unique characteristics. It refers to the intimately shared control where the user’s body is intertwined with the WR and where movement intentions have to shape themselves to the characteristics of the WR. At the same time, the WR may also be linked into systems outside of the physical robot, which may themselves modify robot functioning, thereby adding further complexity to the degree of control given to the user vis-á-vis the machine and other agents.

#### Benefits, Risks and Harms for Self

The core ethical principles of beneficence and non-maleficence (Beauchamp and Childress 2012) are being considered in current practice with WRs, especially in rehabilitation contexts, but merit more complex ethical considerations. One of the primary goals of WRs is to benefit users by replacing, supporting, or enhancing their motor functions. Experts warned about the risk of ‘hyping WRs,’ by overstating their benefits and underplaying their shortcomings, since these remain considerable, including a lack of functional versatility, ease of use, battery life, and intrusive visual appearance. Generally, harm-benefit analyses aim to determine justifiable risks connected to the development and use of WRs (Bissolotti et al. 2018). However, individual values, preferences, and contextual factors significantly impact the assessment of what exactly in each case constitutes a benefit, and how benefits should be balanced against negative aspects of WR use. For some WR users, being able to stand upright in a lower limb exoskeleton or preventing back strain at work constitutes a significant benefit. In contrast, for others, practical shortcomings of WRs in comparison with other mobility or support options, or the fact that their use is employer-mandated may be more prominent. In short, without user involvement, it cannot be taken for granted that WRs deliver benefits that users perceive as such.

Some forms of harm prevention already receive significant attention in the design process of WRs, such as adherence to health and safety laws and regulations, ISO standards, and CE marking. WRs could adversely affect users, for instance, if there are risks of freezing, malfunctioning, toppling over, or if the movements they facilitate or support are physiologically problematic, e.g., when stroke patients are inadvertently assisted with inappropriate compensatory movements. Risks also comprise cybersecurity considerations; data logging, hacking, and malware may lead to physical safety and privacy risks. For future technologies such as brain-computer interfaces that link the robot even more intimately to the human brain, those concerns may be further exacerbated (Nakar et al. 2015).

To do justice to concerns around benefit and harms to users, careful communication to provide meaningful informed consent for WR use is essential to align user expectations with the realities of the WR. Experts mentioned that users might have unrealistic expectations about what the WR will enable them to do (Bissolotti et al. 2018), including potential over-trust of WRs leading to risky use, reported for example for parents of pediatric users (Borenstein et al. 2018).

### Wearable Robots and the Other: Interpersonal Perspectives

#### Ableism and Stigmatization in the Perception of the WR-Supported Body with Disabilities

Experts expressed concerns regarding the perception of WR-supported bodies by others, especially in the rehabilitation domain. Broadly, they were concerned with the image of a ‘standard body’ that a WR is aiming to restore, which often seems to underlie engineering discussions in the rehabilitation field. Through reinforcing a need for ‘fixing’ body functions, WRs shape the understanding of disabilities and medical conditions as purely physical problems in need of a technical solution. Through broader use of WRs, perceptions on normality, disability, and ability might be influenced (Breen 2015; Greenbaum 2015a), insofar as WRs can contribute to a narrower spectrum of accepted bodies, recreating an ‘ideal’ body. Disabled persons might be pressured into using them to achieve perceived ‘normality.’ Such pressure can be interpreted as resulting from the medical model of disability, which has been much criticised in disability studies (Shakespeare et al. 2009). Disabled people have rejected the underlying ableist assumption that disabilities are intrinsically bad, the medical paternalism inherent in the urge to ‘fix’ their bodies and the narrow and often primarily medical and technological range of available solutions. ‘Fixing’ an individual’s abilities might also shift the focus away from accessibility standards for which the disabled community has been advocating (Davis 2012). For example, the possibility of WR-facilitated independent gait could weaken wheelchair users’ claims for public accessibility tools, e.g., ramps and door openers (Klein and Nam 2016). In general, the introduction of WRs poses the question of whether the preferred method to include disabled people is by ‘fixing’ body functions with ‘high-tech gadgetry’ or by constructing accessible environments via social and environmental modifications (Aas and Wasserman 2016). Human well-being and participation in society might be improved more by accepting a disability than by attempting to repair body functions. Interests of society and users need to be negotiated: if society expects disabled people to fix their body functions with WRs, it may become difficult for disabled people without WRs to search for employment and accommodation (Breen 2015).

Being clearly visible on a user’s body, WRs can also create stigma, especially in the rehabilitation domain (Klein and Nam 2016). It has been shown with regard to other assistive technologies, that visibility may lead to reluctance to use the technology (e.g. Söderström and Ytterhus 2010). WR users are potentially perceived differently than people who cannot or choose not to use the technology (Breen 2015). Accordingly, it is not self-evident that using a WR is in every potential user’s interest (Shakespeare and Watson 2019). Given their substantial costs and ELS issues, experts have doubted whether WRs—even when working well—are the best solution to address a person’s impairment (Klein and Nam 2016; Manning 2010).

#### Overestimation and Alienation in the Perception of the WR-Enhanced Professional Body

In industrial and logistical work environments, the use of WRs for the performance of strenuous tasks is increasing. The field also appears set to move into the care sector, targeting WRs to care workers.

In the bioethical enhancement debate, there is substantive disagreement on definitions and appropriate boundary setting regarding enhancement technologies (Parens 2005, 2014); elements of these debates are transferable to the field of WR. The question of where rehabilitation or other health-supporting use ends and non-health related enhancement begins (Greenbaum 2015a, b; Palmerini et al. 2014) also raises potential regulatory issues for WRs, given different regulatory approaches for health and non-health applications (Fosch-Villaronga 2019). In the discussion of enhancement uses of WRs, similar to other enhancements, both exaggeration and underestimation of their likely impacts can be identified. The enhancement discussion often presents the body enhanced by wearable technologies as a ‘cyborg’ (Murata et al. 2017; Pedersen and Mirrlees 2017; Sadowski 2014), as a potentially substantial departure from the unenhanced body. Interpersonally, this perception of significant difference may result in feelings of awe or admiration, or feelings of fear or alienation from such enhanced bodies (Pedersen and Mirrlees 2017).

Such attitudes are likely to impact the perception of workers using WRs. One concern is what a WR-enhanced worker can be reasonably asked to perform, and whether this could potentially lead to additional risks for workers, unequal treatment, and exploitation (Vrousalis 2013). Using WRs may also give rise to greater emotional distance or even feelings of alienation from such workers by others, especially in the contact between WR-enhanced workers and laypersons unfamiliar with WRs. This could be especially problematic for work settings with substantial public-facing interpersonal contact.

#### Care-Giving, Dependencies and Trust

The use of WRs in care-giving might have a particularly noticeable impact on care relationships. Care-giving relationships are characterised by various dependencies; their complexity and ethical significance have been extensively explored in bioethics (e.g. Kittay 2013; Kittay and Feder 2003). WRs can be used in care-giving relationships by care-receivers during their rehabilitation or as a longer-term mobility option, or by caregivers, to support movement execution in certain tasks like lifting patients, so reducing bodily strain. While users may be dependent on the WR to achieve or preserve bodily functions, this may also include dependence on health professionals or carers to help them with donning and using the WR, as well as technicians and engineers responsible for technical support and maintenance.

One significant concern that experts expressed was the potential consequences of misunderstanding how using WRs might impact users’ care needs and their dependency on others. Patients using WRs might be perceived as having increased physical independence and thereby a reduced need for human care. Experts worried this assumption might lead to a reduction of human–human interaction for those users, such as the lowered provision of human-led rehabilitation activities or even a complete replacement of human caregivers (Stahl and Coeckelbergh 2016), not matching their actual care needs. When WR are introduced careful assessment is required of remaining or even newly arising support needs so that patients can continue to receive an adequate level of care. Patients’ WR use may also affect not only users and caregivers but also users’ families, insofar as they are part of intimate, caring networks. This is especially relevant when the recipient of the WR is substantially dependent on their care and their carers’ decision-making, as in the case of children (Bissolotti et al. 2018).

For all WR uses in care settings, the question of trust and trustworthiness arises regarding trust in the devices, trust experienced in the care relationship and wider societal trust in technology, corresponding to concerns identified in the ethical debate on trust (Baier 1986; Jones 2016; O’Neill 2002). The trustworthiness of technology has been emphasised as especially important in the High-Level Expert Group guidance on Trustworthy AI (HLEG AI 2019), which focuses on societal factors of trustworthiness and identifies various design and implementation aspects supporting trustworthiness for devices and applications. Accordingly, the design and implementation of WRs should meet criteria such as those proposed by the HLEG AI. The interpersonal impact of trust also needs to be kept in mind. Users that consider a WR or their care provider trustworthy might more willingly accept the integration of the technology’s use into their care. At the same time, it is essential that such trust is not betrayed and that the WR use does not impair the delivery of care.

### Wearable Robots in Society

#### Technologisation, Dehumanisation and Exploitation

A significant societal concern expressed by some experts was whether the normalisation of WR use on workers’ bodies might make them appear ‘less than human,’ as hybrid machine-optimised bodies to be employed in the service of increased efficiency. In the industry domain, ‘turning workers into machines’ has been connected to the dehumanisation of work and the possible exploitation of workers (Greenbaum 2015a, b). This concern has been brought up more generally regarding worker surveillance practices in some logistical centres and warehouses where workers’ movements are being tightly monitored and optimised with the help of movement trackers and collaborative robots. WRs may intensify this trend through impact on the workers’ bodies themselves.

This raises the concern of exploitation, with WR workers becoming parties to a systematically unfair exchange where they are taken advantage of. Exploitation has been related to concepts such as the appropriation of the worker’s contribution or unfair distribution (Arneson 1981, 2016) and the concepts of vulnerability and domination (Vrousalis 2013, 2018). Imposing the use of WRs on workers might be an act of domination and subjugation in the service of profits, rather than being primarily targeted at workers’ health. Accordingly, if workers’ bodies become more resilient and efficient due to WR use, employers may increase the intensity of expected task performance rather than balancing efficiency benefits holistically against the broader impacts of intensified work practices on workers.

If eventually WR-based enhancement constituted a new norm, disadvantages for non-users might arise, as some experts pointed out, raising the question of individual workers’ and work unions’ rights regarding the introduction and use of WRs in workplaces. This concern might even transfer into the domain of sports, where WR-based enhancements raise questions of fairness, similar to other enhancements in competitive contexts (Greenbaum 2015a).

#### Social Justice, Resources and Access

Access to WRs, especially for rehabilitation use, was one of the most frequently mentioned concerns by experts. Cost is the probably most significant barrier to access; WRs are currently expensive to develop and produce (Cornwall 2015), impeding wide availability for use in rehabilitation. Hence, WRs are often made available to a larger group of users to increase their cost-effectiveness (Bissolotti et al. 2018). WR use is generally not covered by health insurance, evoking questions about who should decide coverage and access criteria (Greenbaum 2015a), including questions of inclusion in prescriptions, fair cost contributions by patients, or the need for rental schemes. Given the current high costs, WRs may benefit only wealthy patients in developed countries, thereby exacerbating social inequality (Greenbaum 2015b; Manning 2010). At the same time, their perception as cutting-edge may potentially marginalise alternative, non-robotic options.

Another potential limitation to access stems from physical requirements. WRs have weight and height restrictions, potentially excluding persons with specific bodyweight, sizes, and shapes (Fosch-Villaronga et al. 2018; Søraa and Fosch-Villaronga 2020). A particular challenge in this context is the need for adaptation for pediatric users with growing and changing bodies (reference removed for review). On the one hand, WRs appear promising, especially for developmental neuromuscular diseases, but on the other hand, the required adaptability of the WR is difficult to achieve.

#### Data Protection and Privacy

Many experts voiced concerns about the generation and use of personal data in WRs. The sensitivity and amount of data processed by WRs are strongly dependent on the WR specifications. WRs may process continuous complex sensor-based data streams as well as additional health-related information and generate user profiles. The physical human-exoskeleton interaction generates data ranging from kinematics, training, exoskeleton performance, ambient data, to a user’s health data. Such information may be collected before or continuously during use from various sensors, e.g., to identify the exoskeleton’s position, torque, and interaction with its environment and to minimise compensatory gait patterns. This information can be presented to the user for general feedback and real-time correction and might be ‘securely saved to the cloud for easier documentation.’ The use of cloud processing may facilitate high-performance real-time data processing, support data statistics and machine-learning analysis (Wang et al. 2019) with advantages such as decreasing the weight of the device, online updating, sharing, and using training data, pilots, and WR data more efficiently.

WR developers process ‘personal data resulting from specific technical processing relating to the physical, physiological or behavioural characteristics of a natural person’. Such biometric data fall under the category of sensitive data of the General Data Protection Regulation (GDPR) and require higher protective measures to ensure the rights of the WR users as data subjects.

Given that WRs provide a comprehensive profile of the user, including physiological characteristics, health status, and associated personal information derived from WR usage, experts were concerned that the user’s personal data might be made available to third parties with interests that may differ from the individual WR user. Data leaks were a general concern. In industrial settings managers might have an interest in close surveillance and data-driven selection of workers. Insurance companies might also find such data useful to ‘help them create more detailed risk profiles on insured workforces and put a lid on ever-rising costs’ (Olson 2014).

Due to WRs’ cyber-physical nature, data security is intimately linked to users’ physical safety due to WRs’ immediate physical effect on their environment and the user (Morante et al. 2015). Given that security and data protection ‘vulnerabilities could allow unauthorized users to remotely access, control, and issue commands to compromised devices, potentially leading to patient harm’ (FDA 2020), vulnerabilities in robotic devices directly fastened to the user’s body deserve particular attention (Fosch-Villaronga et al. 2018; Greenbaum 2015a).

#### Accountability and Responsibility

Responsibility and accountability for robot actions do not just arise regarding individual users, but also on a societal level. One challenge in allocating responsibility is distributed responsibility, i.e., the problem that due to the complexity of various agents’ input into robot development, deployment, and decision-making, various parties have a causal impact on the robot’s output (Floridi 2016), which is even greater in cloud robotics ecosystems (Fosch-Villaronga and Millard 2019). Different parties may share partial responsibility for an outcome. Concerning unintended harmful outcomes, it is essential to have clarity regarding accountability, liability, and litigation for these devices for cases of distributed responsibility (Manning 2010). What makes WRs different from other robotic devices for which this problem has been explored extensively, is the intimate pairing with the human body, where users’ intentions are mediated by the WR and translated into machine movements.

In addition, a potential ‘responsibility gap’ has been identified for systems that have autonomous features, raising the questions whether it is inappropriate to hold human agents responsible for the robot’s autonomous actions (Matthias 2004). However, it has been argued that when the autonomous features are limited and overall designed to be subordinate primarily to human control—such as would be the case for current WRs—responsibility should be fully allocated to humans (Nyholm 2018). How exactly responsibilities should be assigned, e.g., between developers, companies, deploying organisations (such as rehabilitation clinics or industrial production sites) and individual users, remains to be considered in light of the distinctive features of WRs.

Experts also drew attention to developers’ responsibilities for dual-use. Even if WRs are developed for an ethically desirable purpose, such as rehabilitation measures, they might also be used for potentially harmful purposes in different contexts (Greenbaum 2015a). The lines between help and harm, or use and abuse may not always be clear (Howell 2017), and robot technologies are often developed through processes of ‘bi-directional dual-use’ (Nagenborg et al. 2008), frequently moving between civil and military uses (Lin 2010). Experts have warned that it remains unclear how potentially problematic dual uses, once identified, might be prevented and have wondered what responsibilities could be realistically assigned to developers and companies to predict and take proactive measures against them.

#### Legislation and Regulation for WRs

Experts highlighted the overall lack of clarity on the legal frameworks governing WR use. Although a large number of regulatory instruments apply to WRs, emerging technologies tend to fall into an ‘institutional void’ (Hajer 2003), and it can be challenging to understand, both during development and deployment, which regulations apply and how they apply (Fosch-Villaronga 2019). As products, the safety of WRs is regulated ex-ante via the Directive 2001/95/EC on general product safety, and ex-post Directive 85/374/EEC on liability for defective products. To its parts, the Directive 2014/35/EU on low voltage and the electromagnetic compatibility Directive 2014/30/EU may apply. If they collect personal data, the GDPR applies.

WRs for rehabilitation and medical purposes must also follow the binding Medical Device Regulation 2017/745 (MDR) (EU 2017). Compliance with the MDR might also be necessary if WRs are used for non-medical purposes, as its Art. 1.3 states that ‘devices with both a medical and a non-medical intended purpose shall fulfill the requirements applicable to devices cumulatively with an intended medical purpose and those applicable to devices without an intended medical purpose.’ Hence, it is likely that WRs will have to comply with the medical device regulation independently of their intended purpose, since a WR for rehabilitation and for assisting a worker in a factory, for instance, may present similar risks to the user’s health (Fosch-Villaronga 2019; Fosch-Villaronga and Özcan 2019). Developers are encouraged to seek advice from the competent national authorities for medical devices in this respect (CAMD 2020).

WRs could also be categorised as a ‘personal care robot’—a categorization resulting from industry efforts to create an in-between category between the ‘product’ and ‘medical device’ categories (Fosch-Villaronga 2019). The standard ISO 13,482:2014 defines these as ‘service robots that contribute to improving the quality of life of users, excluding medical applications,’ but whereas the MDR is binding, the ISO standard is not. In the context of disability-related uses of WRs used as assistive technologies for mobility, it is also essential to take into account the requirements of the Convention on the Rights of Persons with Disabilities (United Nations 2008) which states principles of respect, inclusion, participation, and accessibility that should inform the process of development and societal implementation of WRs as assistive technologies.

Another complication is the assessment of legal obligations in a time in which technological change is swift, legal instruments are continually being revised, and multiple non-binding resolutions appear (Fosch-Villaronga 2019). For instance, the European Parliament highlighted that designers should ‘draw up design and evaluation protocols and join with potential users and stakeholders when evaluating the benefits and risks of robotics, including cognitive, psychological and environmental ones’ (European Parliament 2017). Increasing attention to the psychological effects arising from the interaction between humans and the technology is also likely to make its way to significant regulations, such as future revisions of the General Product Safety Directive (Fosch-Villaronga 2019).

## Next Steps

As our discussion has shown, ELS concerns for WRs are complex and arise with respect to different application domains and stakeholders. The literature on robot ethics has sometimes been criticised for not being sufficiently connected to actual innovation practices and contexts of use (Stahl and Coeckelbergh 2016). While value-sensitive design approaches (e.g. Borning et al. 2004) have been considered in the literature, their practical impact in the development of robots has been limited. Yet, there are signs of increasing awareness beyond academia of the importance of integrating ELS considerations into the design process of new technologies, as evidenced bythe inclusion of privacy by design requirements in the GDPR, the attention given to the concept of Responsible Research and Innovation (RRI) at the European level, or by the attention given to principles guiding the development of trustworthy AI. Nevertheless, more practical guidance is needed to articulate those general, technology-neutral principles into concrete, actionable recommendations for developers to follow. In order to achieve such transfer, it is essential to pay attention to the specific characteristics of the technologies and their contexts of use, and necessary to identify the specific values and ELS concerns at stake.

The taxonomy of relevant ELS issues for WRs provided here is intended as a starting point not just for further theoretical exploration of the identified concerns but, more importantly, as initial guidance for the ELS-sensitive design and implementation of WRs in different application settings.

To move forward on the integration of ELS issues into WR design and implementation, we consider it essential that particular attention be paid to the following concerns:

### Acknowledge WR ELS Issues as Shared But Distinctive

Addressing ELS issues in WRs will benefit substantially from engagement with the ELS literature on robots and technology in different application domains. The fact that WRs can be used across health, industrial/workplace, recreational and military domains opens up the potential relevance of literature that engages with domain-specific characteristics for the design and deployment of robots in each field. Exploring the significance of discussion from different domains may also allow valuable insights across domains. For WRs in the rehabilitation sector, wider ELS literature on care robots addressing concerns around care, access, disability, and the regulation of healthcare robots will be relevant. For WRs in the workplace, wider ELS literature around human replacement, dehumanisation, exploitation, and workers’ rights should be taken into account. For WRs in the recreational domain, ELS literature on the enhancement debate provides helpful insights. There are also some general cross-cutting concerns, such as vulnerability, user-centredness, safety, or liability.

At the same time, it is essential to do justice to the distinctive characteristics of WRs, particularly the intimate intertwinement of the human body and the WR. This has multiple consequences, from the impact on the user’s subjective experience of their body and its modified functionality, to the interpersonal responses to this intertwinement, the issue of shared agency and responsibility between the user, the WR and the creators and managers of the WR, and the specific bodily risks for users. Exploration of the relevance of these specific characteristics would be essential to achieve ELS-sensitive design and implementation practice for WRs.

### Involve End-Users and Other Stakeholders from the Outset

Due to the different contexts of WR use, various stakeholder groups are involved. Their experiences and views need to be considered to understand practical concerns arising in each context of use. The user perspective, in particular, is essential for understanding ethical challenges, given the unique subjective experience associated with the close intertwinement of the user’s body and WR. More empirical research is needed to understand user experiences and attitudes towards the use of WRs among their main user groups, especially concerning persons with disabilities and industry workers. Patient organisations and workers unions could help inform understanding of those contexts of use and users’ experiences in these settings.

In addition, it is also crucial to capture the interpersonal dimension of perceiving and engaging with WR users in different application contexts by exploring perceptions and attitudes of those interacting with WR users in family, professional care, industrial or recreational settings. Concerning the broader social dimension, it would be desirable to understand trends and the current practical reach of WRs. Qualitative social science methodologies should be used for in-depth case studies of real WR use in different contexts, that are sensitive to the embeddedness of WRs in context-specific social relations. Without a solid knowledge of the realities of WR use in these three dimensions, an essential component of ELS-sensitive design would be missing.

### Make Applicability of Existing Legislative and Regulatory Framework More Explicit

The legislative and regulatory situation regarding WRs is complex, insofar as while there are numerous relevant pieces of legislation and regulation addressing various aspects of WR use, there is a lack of both comprehensive and sufficiently specific laws and regulations for these robots. The current pieced-together framework brings about uncertainties for WR designers and those deploying them regarding legal boundaries between different types of use, liability issues, appropriate levels of safety requirements, and users’ rights. Accordingly, questions remain regarding which obligations developers and those deploying the WR for others have, and what legal consequences are in place in case of non-compliance (Stilgoe et al. 2013; Fosch-Villaronga 2019). A more unified framework could bring legal certainty, improve safety, and acceptance (Fosch-Villaronga and Ozcan 2019). In the absence of newly developed legal and regulatory structures, however, more clarity on how existing legal and regulatory instruments apply should be provided for WR developers and those deploying them.

## Conclusion

In this paper, we have shown that despite their evident complexity, there is comparatively little attention given to ELS reflection about Wearable Robots, and have argued that this is a gap that deserves to be filled, both for theoretical and practical reasons. Theoretically, WR technology raises specific issues regarding the consequences of the close intertwinement of the machine and the human body that differ from ELS concerns regarding other types of robots. ELS engagement with WRs may benefit specifically from taking on board considerations from disability studies, care ethics, and the enhancement debate.

Concerning the practical significance of engaging with ELS aspects of WR, we consider our proposal of a taxonomy of relevant concerns to be a useful starting point for identifying context- and stakeholder-specific considerations that could improve the ELS-sensitive management of potential challenges in the development and deployment of WRs as part of a value-led design process. To do justice to these concerns, we have argued that several additional conditions need to be in place. Reflection on ELS issues in WRs would benefit from engagement with established areas of consideration in other fields of robot ethics and technology ethics, but careful attention needs to be paid to the complexity of the implications of the distinctive features of WRs. An empirical investigation of WR user and other stakeholder perspectives in real-life WR use settings is so far significantly underrepresented in the debate. It should be more actively and systematically sought out concerning different application settings and the three dimensions—subjective, interpersonal, and social—proposed here.

Finally, there is a need to clarify the legal and regulatory landscape governing WRs, especially the interactions and potential fault lines between the different instruments, to allow developers to navigate those requirements. We hope that future contributions to the debate will effectively address these gaps and move forward towards ELS-sensitive design and deployment.
